# Impact of Systemic Ventricular Morphology on Age-Adjusted (zlog-)NT-proBNP in Children with Univentricular Hearts

**DOI:** 10.1007/s00246-025-03898-2

**Published:** 2025-06-03

**Authors:** Jonas Palm, Carolin Niedermaier, Stefan Holdenrieder, Georg Hoffmann, Frank Klawonn, Jürgen Hörer, Masamichi Ono, Peter Ewert

**Affiliations:** 1 Department of Pediatric Cardiology and Congenital Heart Defects, German Heart Center, TUM University Hospital, Munich, Germany; 2 Department for Congenital and Pediatric Heart Surgery, German Heart Center, TUM University Hospital, Munich, Germany; 3 Division for Congenital and Pediatric Heart Surgery, University Hospital Großhadern, Ludwig-Maximilians University, Munich, Germany; 4 Institute of Laboratory Medicine, German Heart Center of the Technical University Munich, Munich, Germany; 5 Helmholtz Center for Infection Research, Brunswick, Germany; 6 Institute for Information Engineering, Ostfalia University of Applied Sciences, Wolfenbuttel, Germany

**Keywords:** NT-proBNP, Zlog, Single ventricle, Morphology, Fontan

## Abstract

As a marker of cardiac wall stress, NT-proBNP offers high prognostic and diagnostic potential in patients with a functional single ventricle (fSV). Its levels depend on both age and stage of palliation. However, the impact of systemic ventricular morphology on this biomarker remains unclear. Children undergoing staged palliation, i.e. systemic-to-pulmonary shunt (SPS), ductal stenting (DS) and/or pulmonary artery banding (PAB) as stage 1, bidirectional cavopulmonary shunt (BCPS) as stage 2 or extracardiac total cavopulmonary connection (TCPC) as stage 3 at our institution between 2011 and 2023 were identified. Those, who had NT-proBNP determined at most 7 days before intervention or surgery were included. Furthermore, patients at least 6 months after TCPC with ambulatory measured NT-proBNP were enrolled. NT-proBNP levels were evaluated using its age-adjusted z-score ("zlog-NT-proBNP"), allowing comparison irrespective of the distinctive physiological decline with age. Overall, 618 children met the eligibility criteria. Thereof, 356 patients had a systemic right ventricle (SRV) and 262 a systemic left ventricle (SLV). At each stage of palliation, age-adjusted zlog-NT-proBNP was significantly higher in patients with an SRV compared to an SLV: before SPS/DS/PAB (median 3.43 vs 2.62, p < 0.001); before BCPS (median 3.33 vs 2.04, p < 0.001); before TCPC (median 1.50 vs 0.66, p < 0.001); and after TCPC (median 1.62 vs 0.81, p < 0.001). Systemic ventricular morphology highly affects (zlog-)NT-proBNP levels in fSV patients at each stage of palliation. When interpreting NT-proBNP levels in these patients, clinicians and future studies should take into account that children with an SRV reveal higher NT-proBNP levels than those with an SLV.

## Introduction

Patients with a functional single ventricle (fSV) represent a highly heterogeneous and challenging population within congenital heart defects (CHD). With an estimated incidence of 4–8/10,000 live births, they account for approximately 7.7% of all CHDs [1]. Irrespective of the individual morphology, fSV patients share the existence of an anatomically or functionally solitary ventricle [2], ensuring perfusion of both pulmonary and systemic circulation. Since the prognosis of the different entities is highly unique but extremely poor in general, infants with fSV are palliated in up to three stages, in which 15-year survival rates of up to 95% are possible today [3–6]. The palliative strategy consists of three consecutive surgical steps with distinctive hemodynamic consequences:

- Stage 1 (early after birth): balancing pulmonary blood flow by a systemic-to-pulmonary shunt (SPS), ductal stent (DS) and/or pulmonary artery banding (PAB).
- Stage 2 (at 3 to 6 months of age): reducing cardiac output by a bidirectional cavopulmonary shunt (BCPS).
- Stage 3 (first years of life): eliminating cyanosis by separating pulmonary and systemic circulation by a total cavopulmonary connection (TCPC).

As a marker of myocardial volume and pressure load, N-terminal pro-B-type natriuretic peptide (NT-proBNP) is a highly promising tool to assess systemic ventricular strain easily and objectively in these patients. However, physiological NT-proBNP dynamics are characterized by exceptionally high concentrations during infancy and an exponential decline until adulthood [7, 8]. Thus, the surgical steps of staged palliation fall precisely in a period of time when NT-proBNP age dynamics are particularly pronounced and different reference intervals (RIs) apply at each stage, impeding its use as a course parameter. A reliable solution to this obstacle is provided by the zlog value of NT-proBNP (“zlog-NT-proBNP”), which relates the measured NT-proBNP concentration to age-specific RI analogous to a z-score [9, 10]. It favourably predicts major adverse cardiac events (MACE) in children with congenital heart disease and is prognostically superior to absolute NT-proBNP concentrations [11].

However, there are other factors besides age that strongly influence NT-proBNP in children with fSV, such as the stage of palliation [12]. In this context, the impact of the systemic ventricular morphology so far remains unclear. From a pathophysiological point of view, it seems plausible that patients with a systemic right ventricle (SRV) have higher myocardial stress than patients with a systemic left ventricle (SLV) due to the fibromuscular structure and different physiological function, resulting in higher NT-proBNP levels. Therefore, in the following study, we evaluated NT-proBNP by means of its zlog value for age-independent comparison during all stages of palliation, taking into account the morphology of the systemic ventricle.

## Methods

### Study Population

At the German Heart Center Munich, a tertiary center for pediatric heart disease, all children under 18 years of age with a functional or anatomical single ventricle who underwent systemic-to-pulmonary shunt (SPS) including the Norwood procedure, ductal stenting (DS) and/or pulmonary artery banding as stage 1 palliation (S1P), bidirectional cavopulmonary shunt (BCPS) as stage 2 palliation (S2P), or total cavopulmonary connection (TCPC) as stage 3 palliation (S3P) between January 1, 2011, and December 31, 2023, were identified. Of these patients, those who had at least one zlog-NT-proBNP measurement within the seven preoperative days before any of the three palliative procedures (SPS/DS/PAB, BCPS, or TCPC) were included in the analyses. In addition, children with a (zlog-)NT-proBNP measurement at least 6 months after Fontan completion by (extracardiac) TCPC who presented to our outpatient clinic for routine follow-up were included. For this group, however, we included all patients under 18 years of age with ambulatory NT-proBNP measurement and (extracardiac) TCPC, even if the surgery was performed before 2011. Patients who underwent TCPC conversion surgery or other types than extracardiac TCPC were excluded from this study.

### Baseline Characteristics

For each stage and patient, clinical data such as age, sex, main cardiac diagnosis, systemic ventricular morphology, concomitant malformations, type and date of surgical procedures were obtained retrospectively from medical records, as were preoperative height and weight (only patients before TCPC).

### Measurement of NT-proBNP and Calculation of Age-Adjusted zlog Values

For several years, the preoperative determination of NT-proBNP has been routinely performed on admission at our institution. Blood samples were collected using standard techniques. Concentrations were determined using the Roche Diagnostics Elecsys® proBNP II assay (monoclonal antibodies) on a cobas® e 411 system. Concentrations above the assay’s upper limit of 35,000 ng/L have been routinely repeated and extrapolated after dilution since 2021 (before that time upon request). Corresponding age-adjusted zlog values of NT-proBNP (“zlog-NT-proBNP”) were calculated as previously described (online calculator available here) [9]. Analogous to a common z-score, zlog-NT-proBNP indicates the number of standard deviations by which the measured NT-proBNP concentration is above or below the age-specific mean on a logarithmic scale [9, 10]. Therefore, its reference interval age-independently ranges between − 1.96 and + 1.96.

### Statistical Analysis

Categorical variables are presented as absolute numbers with percentages, and compared using chi-square test. Continuous variables were expressed as mean with standard deviation (SD) and compared using the student’s *t* test if approximately normally distributed; otherwise, median with interquartile ranges (IQR) was reported and the Mann–Whitney *U* test was used for group comparison. The partially matched Wilcoxon test was used to compare zlog-NT-proBNP levels between stages within different morphological groups, as some patients had measurements at multiple stages. A p-value < 0.05 was considered significant for two-sided tests. Statistical analyses were done using R version 4.2.0 and the Statistical Package for the Social Sciences (SPSS) version 28 for Windows (IBM, Ehningen, Germany).

## Results

### Baseline Characteristics and Diagnoses

**Fig. 1:**
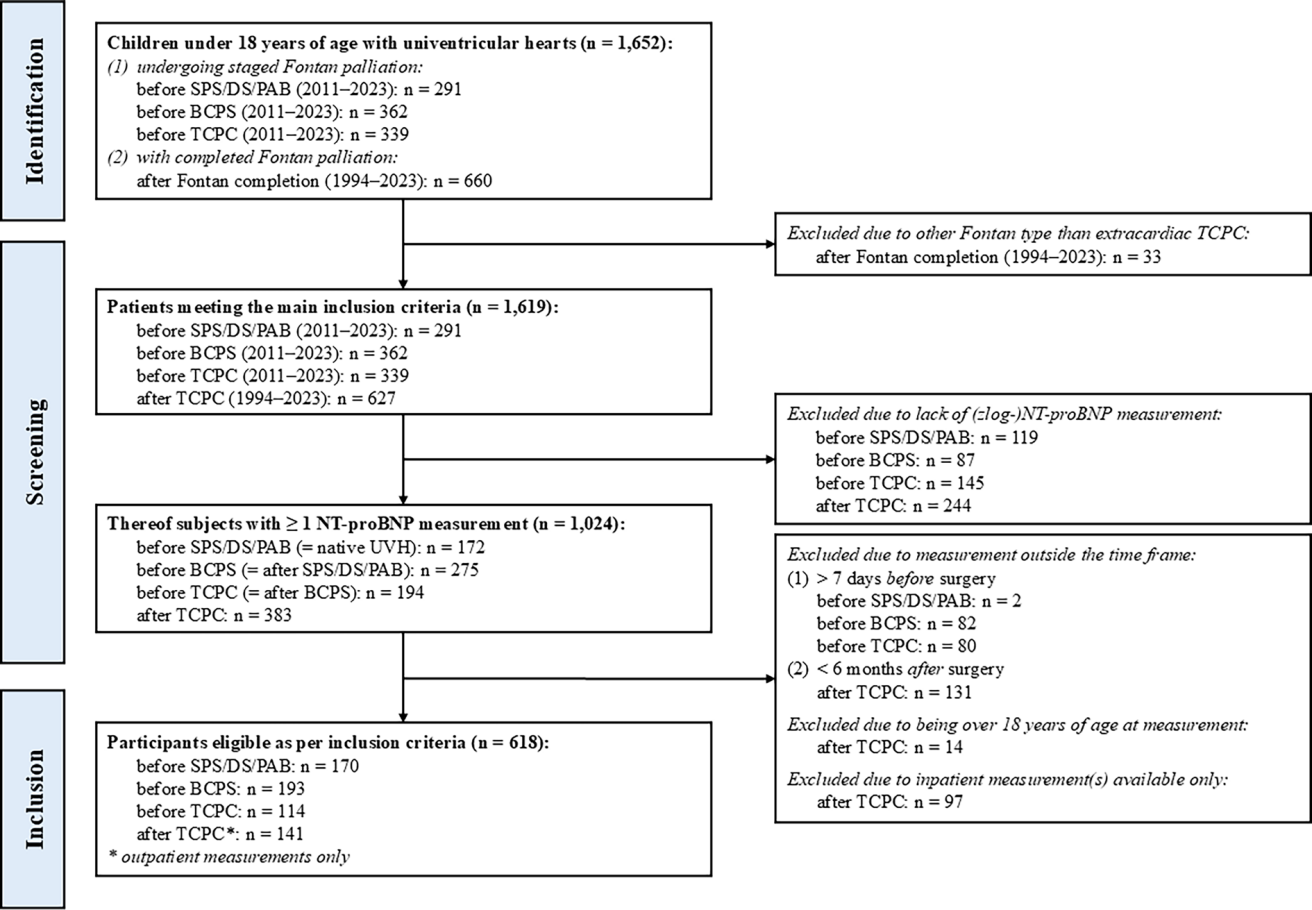
Flow diagram of patient selection. *SPS* systemic-to-pulmonary shunt, *DS* ductal stent, *PAB* pulmonary artery banding, *BCPS* bidirectional cavopulmonary shunt, *TCPC* total cavopulmonary connection

A total of 618 children with fSV meeting the inclusion criteria were enrolled (Fig. 1). Of these, 389 (63%) were male and 229 (37%) were female. 170 children (median age 8 [6–15] days, n = 111 with SRV) underwent SPS, DS and/or PAB as the first stage, 193 children (median age 117 [99–146] days, n = 111 with SRV) underwent BCPS as the second and 114 children (median age 2.2 [1.9–2.6] years, n = 76 with SRV) underwent extracardiac TCPC as third and final step of palliation. In addition, 141 patients with ambulatory measured NT-proBNP were identified after (extracardiac) TCPC (median age 10.6 [7.7–13.2] years, n = 76 with SRV). Further patient characteristics are shown in Table 1.

**Table 1:** Baseline characteristics

Characteristic	No. (%) or median (IQR)
Subjects—no	618
Age—years	
Median (IQR)	0.4 (0.1–3.3)
Range	0.0–18.0
Sex—no. (%)	
Male	389 (62.9%)
Female	229 (37.1%)
Height—cm	
Median (IQR)	60 (52–76)
Range	42–162
Weight—kg	
Median (IQR)	4.9 (3.4–8.5)
Range	1.8–46.0
Condition—no	
Native functional SV (before SPS/DS/PAB)	170 (27.5%)
Stage-1-Palliation (before BCPS)	193 (31.2%)
Stage-2-Palliation (before TCPC)	114 (18.4%)
Stage-3-Palliation (after TCPC)	141 (22.8%)
Laboratory data—median (IQR)	
NT-proBNP (age-adjusted)—zlog value	2.09 (1.10–3.33)
NT-proBNP (absolute)—ng/L	1.290 (199–7.250)
Time between NT-proBNP and surgery—median (IQR)	
Preoperative measurement (before SPS/DS/PAB, BCPS or TCPC)	2 (1–3) days
Postoperative measurement (after TCPC)	8.4 (4.8–10.8) years
Age at surgery—days/years	
Systemic-to-pulmonary shunt (SPS), ductal stent (DS) or PAB	8 (6–15) days
Bidirectional cavopulmonary shunt (BCPS)	117 (99–146) days
Total cavopulmonary connection (TCPC)	2.2 (1.9–2.6) years
Primary diagnosis—no. (%)	
Congenitally corrected transposition of the great arteries (ccTGA)	22 (3.6%)
Double inlet left ventricle (DILV)	100 (16.2%)
Double inlet right ventricle (DIRV)	4 (0.6%)
Double outlet right ventricle (DORV)	34 (5.5%)
Hypoplastic left heart syndrome (HLHS)	221 (35.8%)
Pulmonary atresia with intact ventricular septum (PA-IVS)	32 (5.2%)
Tricuspid atresia (TA)	74 (12.0%)
Unbalanced atrioventricular septal defect (unAVSD)	33 (5.3%)
Others: (complex) functional single ventricles (fSV)	98 (15.9%)
Associated cardiac anomaly—no. (%)	
(Hemi-)azygos vein	15 (2.4%)
Anomalous pulmonary vein return (APVR)	31 (5.0%)
Aortic anomalies (hypoplasic arch, CoA, IAA)	324 (52.4%)
Common atrioventricular valve (CAVV)	71 (11.5%)
Dextrocardia	37 (6.0%)
Heterotaxy	35 (5.7%)
Persistent left superior vena cava (PLSVC)	68 (11.0%)
Trans-/malposition of the great arteries (TGA, MGA)	225 (36.4%)
Systemic ventricular morphology	
Systemic left ventricle (SLV)	262 (42.4%)
Systemic right ventricle (SRV)	356 (57.6%)

*SPS* systemic-to-pulmonary shunt, *DS* ductal stent, *PAB* pulmonary artery banding, *BCPS* bidirectional cavopulmonary shunt, *TCPC* total cavopulmonary connection

The most common diagnoses were hypoplastic left heart syndrome (HLHS) in 221 (36%) patients, double inlet left ventricle (DILV) in 100 (16%), and tricuspid atresia (TA) in 74 (12%). Consistent with the majority of patients with HLHS, the most frequent associated malformation was aortic (arch) anomaly (n = 324), followed by trans- or malposition of the great arteries (n = 225) and persistent left superior vena cava (n = 68). Fewer cases were associated with dextrocardia (n = 37) or heterotaxy syndrome (n = 35).

### Patient Characteristics According to Systemic Ventricular Morphology

A total of 356 (58%) of patients had an SRV, matching HLHS as the most common diagnosis. Accordingly, aortic (arch) anomalies were more prevalent in patients with SRV (p < 0.001), while trans-/malposition of the great arteries was more frequent in subjects with SLV (p < 0.001) (Table 2). No differences were noticed regarding (hemi-)azygos continuity (p = 0.85), dextrocardia (p = 0.65) or heterotaxy (p = 0.06). Patients underwent each stage at a similar age (S1P: p = 0.08; S2P: p = 0.21; S3P: p = 0.62). Regarding length/height and weight before surgery, a significant difference between the morphological groups was found only for BCPS (median 60 cm, 5.0 kg in SRV patients compared to 62 cm, 5.4 kg in SLV patients; p = 0.03 for length/height and p = 0.004 for weight).

**Table 2:** Patient characteristics according to systemic ventricular morphology

Characteristic	Systemic left ventricle (SLV)	Systemic right ventricle (SRV)	p-value
Subjects—no. (%)	262 (42.4%)	356 (57.6%)	
(Age-adjusted) zlog-NT-proBNP—median (IQR)	1.56 (0.71–2.59)	2.72 (1.61–3.70)	< 0.001
Age—years			0.016
Median (IQR)	0.6 (0.2–4.2)	0.3 (0.0–2.6)	
Range	0.0–18.0	0.0–17.0	
Sex—no. (%)			0.007
Male	149 (56.9%)	240 (67.4%)	
Female	113 (43.1%)	116 (32.6%)	
Length/height—median (IQR)			
Before SPS/DS/PAB—cm	51 (49–53)	51 (49–53)	0.39
Before BCPS—cm	62 (59–65)	60 (58–63)	0.03
Before TCPC—cm	87 (82–91)	87 (82–92)	0.68
Weight—median (IQR)			
Before SPS/DS/PAB—kg	3.2 (3.0–3.8)	3.1 (2.8–3.4)	0.08
Before BCPS—kg	5.4 (4.9–6.2)	5.0 (4.3–5.7)	0.004
Before TCPC—kg	123 (105–152)	112 (97–142)	0.68
Age at surgery—median (IQR)			
SPS, DS or PAB—days	9 (6–22)	8 (6–13)	0.08
Bidirectional cavopulmonary shunt (BCPS)—days	123 (105–152)	112 (97–142)	0.21
Total cavopulmonary connection (TCPC)—years	2.0 (1.8–2.5)	2.3 (1.9–2.7)	0.62
Current stage of palliation—no. (%)			
Native functional SV (before SPS/DS/PAB)	59 (22.5%)	111 (31.2%)	
Stage-1-Palliation (before BCPS)	82 (31.3%)	111 (31.2%)	
Stage-2-Palliation (before TCPC)	56 (21.4%)	58 (16.3%)	
Stage-3-Palliation (after TCPC)	65 (24.8%)	76 (21.3%)	
NT-proBNP within age-dependent RI—no. (%)			< 0.001
zlog-NT-proBNP ≤ + 1.96 (normal)	165 (63.0%)	127 (35.7%)	
zlog-NT-proBNP > + 1.96 (elevated)	97 (37.0%)	229 (64.3%)	
Primary diagnosis—no. (%)			< 0.001
Congenitally corrected transposition of the great arteries (ccTGA)	10 (3.8%)	12 (3.4%)	
Double inlet left ventricle (DILV)	100 (38.2%)	0 (0%)	
Double inlet right ventricle (DIRV)	0 (0%)	4 (1.1%)	
Double outlet right ventricle (DORV)	0 (0%)	34 (9.6%)	
Hypoplastic left heart syndrome (HLHS)	0 (0%)	221 (62.1%)	
Pulmonary atresia with intact ventricular septum (PA-IVS)	32 (12.2%)	0 (0%)	
Tricuspid atresia (TA)	74 (28.2%)	0 (0%)	
Unbalanced atrioventricular septal defect (UAVSD)	9 (3.4%)	24 (6.7%)	
Others: (complex) functional single ventricles (fSV)	36 (13.7%)	61 (17.1%)	
Associated cardiac anomaly—no. (%)			
(Hemi-)azygos vein	6 (2.3%)	9 (2.5%)	0.85
Anomalous pulmonary vein return (APVR)	5 (1.9%)	26 (7.3%)	0.02
Aortic anomalies (hypoplasic arch, CoA, IAA)	64 (24.4%)	260 (73%)	< 0.001
Common atrioventricular valve (CAVV)	23 (8.8%)	48 (13.5%)	0.07
Dextrocardia	17 (6.5%)	20 (5.6%)	0.65
Heterotaxy	7 (2.7%)	28 (7.9%)	0.06
Persistent left superior vena cava (PLSVC)	25 (9.5%)	43 (12.1%)	0.32
Trans-/malposition of the great arteries (TGA, MGA)	129 (49.2%)	96 (27%)	< 0.001

*SPS* systemic-to-pulmonary shunt, *DS* ductal stent, *PAB* pulmonary artery banding, *BCPS* bidirectional cavopulmonary shunt, *TCPC* total cavopulmonary connection

Zlog-NT-proBNP levels were significantly higher in patients with a systemic right ventricle compared to those with a left ventricle (2.72 [1.61—3.70] vs 1.56 [0.71—2.59]; p < 0.001) and patients with an SLV were significantly more often within age-dependent NT-proBNP reference intervals (i.e. zlog-NT-proBNP ≤ 1.96; 63.0% of SLV patients versus 35.7% of SRV patients). However, patients with an SRV were significantly younger (median age 0.3 vs 0.6 years; p = 0.007), as they represented a larger proportion within the “native fSV” (before SPS/DS/PAB) group (31.2% of SRV patients versus 22.5% of SLV patients). Further details are depicted in Table 2.

### Zlog-NT-proBNP in Different Stages of SV Palliation

**Fig. 2:**
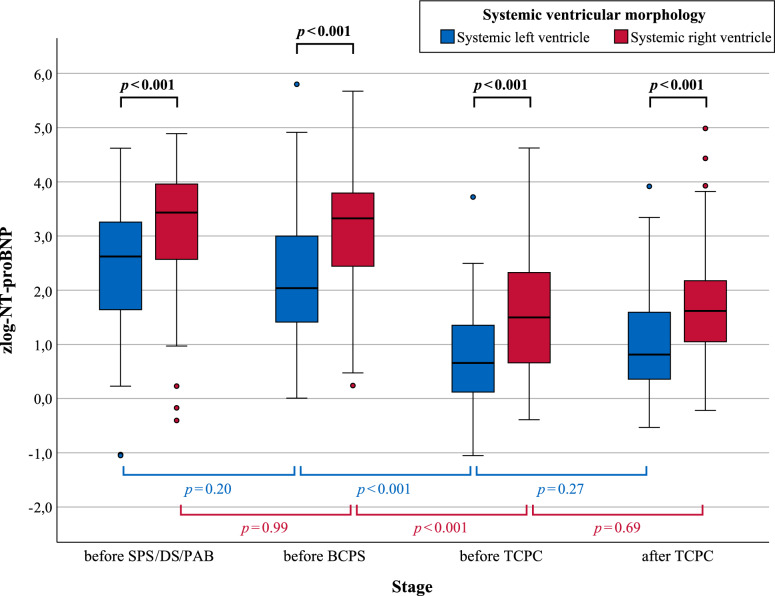
Age-adjusted (zlog-)NT-proBNP levels at different stages of palliation considering systemic ventricular morphology. Age-adjusted zlog-NT-proBNP levels during staged palliation were significantly higher in patients with a systemic right ventricle compared to those with a left one at each stage (see Table 3). A Mann–Whitney U test was used to compare SLV and SRV within each stage (all p < 0.001). To evaluate the effect of SPS/DS/PAB, BCPS, and TCPC within the morphological groups, a partially matched Wilcoxon test was carried out due to partial patient overlap (i.e. comparison of before versus after the respective surgery). *SPS* systemic-to-pulmonary shunt, *DS* ductal stent, *PAB* pulmonary artery banding, *BCPS* bidirectional cavopulmonary shunt, *TCPC* total cavopulmonary connection

Patients with an SRV had significantly higher zlog-NT-proBNP levels at each stage of palliation (all p < 0.001; Table 3, Fig. 2). However, within the morphological groups, only BCPS led to a statistically significant decrease in zlog-NT-proBNP levels, which was evident in both children with SLV (median 2.04 before BCPS and 0.66 thereafter; p < 0.001 for partially matched Wilcoxon test) and in those with SRV (median 3.33 before BCPS and 1.50 thereafter; p < 0.001). In contrast, no significant changes were observed when comparing levels “before SPS/DS/PAB” with “before BCPS” (i.e. evaluating the effect of S1P; p = 0.20 for SLV and p = 0.99 for SRV), or “before TCPC” with “after TCPC” (i.e. evaluating the effect of TCPC; p = 0.27 for SLV and p = 0.69 for SRV).

**Table 3:** Comparison of (zlog-)NT-proBNP levels in patients with systemic left (SLV) vs right (SRV) ventricle with respect to the stage of palliation

Current stage of palliation	zlog-NT-proBNP – *log* SD		
	*SLV*, *median (IQR)*	*SRV,* *median (IQR)*	p-value
Native functional SV (before SPS, DS or PAB)	2.62 (1.64–3.27)	3.43 (2.52–3.97)	< 0.001
Stage-1-Palliation (before BCPS)	2.04 (1.41–3.00)	3.33 (2.39–3.79)	< 0.001
Stage-2-Palliation (before TCPC)	0.66 (0.12–1.35)	1.50 (0.66–2.33)	< 0.001
Stage-3-Palliation (after TCPC)	0.81 (0.36–1.59)	1.62 (1.05–2.18)	< 0.001

Patients with a systemic right ventricle (SRV) had significantly higher zlog-NT-proBNP levels at each stage of palliation (all p < 0.001; p values refer to the difference of zlog-NT-proBNP between children with SRV and SLV). The most pronounced decline occurred with BCPS due to volume unloading of the systemic ventricle (median difference − 1.38 [log SD] for SLV and − 1.83 for SRV, both p < 0.001). *SPS* systemic-to-pulmonary shunt, *DS* ductal stent, *PAB* pulmonary arter banding, *BCPS* bidirectional cavopulmonary shunt, *TCPC* total cavopulmonary connection

**Fig. 3:**
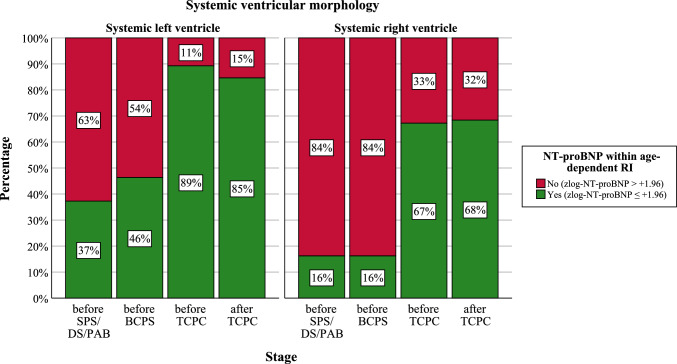
Normal vs elevated (zlog-)NT-proBNP levels according to stage and systemic ventricular morphology. In both the SRV and SLV group, (zlog-)NT-proBNP levels decreased with each surgical step performed. This is also reflected by an increase of patients with NT-proBNP concentrations within age-matched reference intervals (green; zlog-NT-proBNP ≤  + 1.96) and a decline of those with elevated values (red; zlog-NT-proBNP >  + 1.96). *SPS* systemic-to-pulmonary shunt, *DS* ductal stent, *PAB* pulmonary artery banding, *BCPS* bidirectional cavopulmonary shunt, *TCPC* total cavopulmonary connection

After TCPC (stage 3), more than twice as many patients with SRV were above the 97.5th percentile of the age-dependent NT-proBNP concentration (i.e. zlog-NT-proBNP >  + 1.96) than those with SLV (32% vs 15%). Figure 3 illustrates the proportion of patients who are within the age-dependent NT- proBNP reference interval, depending on the stage of palliation.

## Discussion

To date, this is the largest cohort to investigate the impact of systemic ventricular morphology on B-type natriuretic peptides (BNP and NT-proBNP) in children with fSV. Furthermore, it is the first study to compare NT-proBNP levels in children with SLV to those with SRV at each stage of palliation, taking into account age-dependent reference intervals as a major confounder.

### Comparison to Previous Studies

The impact of systemic ventricle morphology on BNP and its inactive metabolite NT-proBNP during staged palliation is discussed controversially [13–20]. After completed Fontan palliation most studies did not find a difference between morphological right and left systemic ventricles for BNP [13–15]. In contrast, higher NT-proBNP levels were demonstrated for SRV both before and after completed staged palliation [17, 18], although the study by Lechner et al. did not reach statistical significance due to an insufficient number of patients [17]. Presumably, this discrepancy between both biomarkers is due to the different half-lives and the age dynamics which are much more pronounced in NT-proBNP [21]. Before BCPS, Holmgren et al. did not find a difference in terms of BNP concentration [16], whereas Butts et al. (n = 173) demonstrated higher values in children with systemic right ventricle [19]. A further study by Eerola and colleagues investigating NT-proBNP before BCPS and before shunt (n = 19) was too small to make a valid statement regarding the influence of systemic ventricle morphology [20].

In conclusion, although the data are somewhat clearer for BNP, they cannot be extrapolated to NT-proBNP, since clear ambiguities exist due to the different (patho)physiology. Thus, our study provides clarity regarding the question whether systemic ventricular morphology has an influence on (zlog-)NT-proBNP. Moreover, and of particular relevance, age was eliminated as an important confounding factor using its age-adjusted zlog value.

### NT-proBNP in Patients with Systemic Right Versus Left Ventricles

In our cohort, patients with a systemic right ventricle had significantly higher zlog-NT-proBNP levels than those with a systemic left ventricle at each stage (all p < 0.001). There are several possible explanations for this phenomenon: In general, a morphologically right ventricle seems less suitable to supply the systemic circulation with its intrinsically higher afterload due to its anatomical shape and contraction pattern [22, 23]. Potential sequelae are ventricular dilatation resulting in a higher grade AV valve regurgitation (which in turn increases volume load on the fSV) and impaired systemic ventricular function [23, 24]. Additionally, patients with HLHS and its variants often undergo surgery with cardiac arrest and hypothermia during the first stage of palliation, which in turn can lead to myocardial ischemia and deterioration of fSV function [25]. Presumably, all of these factors combined ultimately lead to an increased burden on the right systemic ventricle, which is reflected in our cohort as significantly higher zlog-NT-proBNP levels in the SRV group during all stages.

### Effect of S1P, S2P and S3P on (Zlog-)NT-proBNP

In our study, balancing the pulmonary and systemic circulation as part of the first step of palliation did not achieve a significant decrease in zlog-NT-proBNP values (p = 0.20 for SLV, p = 0.98 for SRV). However, further studies are needed to investigate the effect of the type of surgery (especially the influence of ischemia and hypothermia during the Norwood procedure) and the preoperative state of lung perfusion (underperfusion versus overperfusion). Nevertheless, 46% of SLV patients before BCPS had NT-proBNP values within age-dependent reference intervals, while the proportion in the SRV group was only 16%. This suggests that a morphologically left systemic ventricle copes significantly better with the volume overload that exists up to BCPS.

With BCPS, consistent with previous studies, the systemic ventricle experiences significant volume unloading, as upper extremity venous blood is passively directed to the lungs without recirculating via the fSV [19, 26]. This relieves the systemic ventricle, resulting in a distinct decrease of (zlog-)NT-proBNP (p < 0.001 for both SLV and SRV). Nevertheless, (zlog-)NT-proBNP remain elevated in 67% of SRV patients after BCPS (i.e. in the “before TCPC” group), whereas levels normalize in the majority (89%) of patients with an SLV (Table 3, Fig. 3).

With TCPC, technically speaking, the systemic ventricle undergoes complete volume relief due to the connection of the IVC with the pulmonary artery as part of the TCPC [27–29]. However, no significant changes were observed regarding (zlog-)NT-proBNP levels before compared to after TCPC (p = 0.27 for SLV and p = 0.69 for SRV). Thus, the volume load remaining after BCPS does not seem to play a significant hemodynamic role, which is also indicated by the unchanged (zlog-)NT-proBNP levels after TCPC. Consequently, complete circulatory separation by TCPC is less of a relief to the systemic ventricle but primarily improves oxygenation.

### Outlook

Our study provides clarity regarding the extent to which systemic ventricular morphology influences NT-proBNP in children with a fSV. In this context, palliation stage also plays a decisive role. The stage- and morphology-dependent values of zlog-NT-proBNP provided may serve as a valuable guide for healthcare professionals by allowing an indicative assessment of NT-proBNP levels within this population for the first time. Future studies aiming, for example, to explore the prognostic role of NT-proBNP in these patients or to determine associations with pathological conditions such as severity of AV valve regurgitation and impaired fSV function should make appropriate adjustments for systemic ventricular morphology.

### Study Limitations

The study was limited by its retrospective and single-center design. Since clinical status and potential pathologies such as AV valve regurgitation or impaired systemic ventricular function were not considered within this study, the zlog-NT-proBNP levels provided may not serve as typical reference intervals, as they are based on the entire patient population. In addition, although NT-proBNP is part of our admission laboratory, it cannot be completely excluded that some measurements were conducted upon clinical indication. Prior to 2021, NT-proBNP concentrations above the assay’s upper limit of 35,000 ng/L usually were repeated and extrapolated after dilution upon request (see Methods section). Therefore, maximum zlog values were limited in some patients, especially in neonates with SRV prior to SP shunt surgery. However, this means that the values obtained are more likely to be underestimated, possibly increasing the difference between SRV and SLV.

## Conclusions

At each stage, before, during, and after single ventricle palliation, (zlog-)NT-proBNP levels were significantly higher in patients with an SRV compared to those with an SLV. While the vast majority of patients with a systemic left ventricle already revealed NT-proBNP levels within age-dependent reference intervals after BCPS, the proportion of children with a systemic right ventricle with elevated concentrations was two to three times higher. In conclusion, systemic ventricular morphology profoundly affects (zlog-)NT-proBNP and therefore warrants consideration when interpreting or investigating this biomarker in children with fSV.

## Data Availability

No datasets were generated or analysed during the current study.

## Abbreviations

**BCPS**: Bidirectional cavopulmonary shunt
**BNP**: B-type natriuretic peptide
**NT-proBNP**: N-terminal pro-B-type natriuretic peptide
**RI**: Reference interval
**SP shunt**: Systemic-to-pulmonary shunt
**SLV**: Systemic left ventricle
**SRV**: Systemic right ventricle
**TCPC**: Total cavopulmonary connection
**SV**: Single ventricle
**fSV**: Functional single ventricle
**zlog-NT-proBNP**: Zlog value of NT-proBNP
